# A rare case of adult-onset spastic paraparesis associated with Klinefelter syndrome

**DOI:** 10.1186/s12883-024-03525-2

**Published:** 2024-01-15

**Authors:** Louise Adams, Jan De Bleecker

**Affiliations:** Department of Neurology, Ghent University Hospital, Corneel Heymanslaan 10, Ghent, 9000 Belgium

**Keywords:** Spastic paraparesis, Klinefelter syndrome

## Abstract

**Report:**

The rare association of Klinefelter syndrome and the clinical presentation of a late onset chronic progressive spastic paresis.

**Clinical Presentation and Genetics:**

An infertile, 61-year-old man, presented with late adult onset of gait problems, deep muscle pain, and bladder problems. He presented for the first time, years after onset with a spastic paraparesis with high arched feet. His parents had already died, but the patient described high arched feet with his mother. There is no further certain information about the parents. After thorough investigation, an additional X chromosome was found, whereafter the diagnosis of Klinefelter syndrome could be made. Other acquired and genetic causes for spastic paraparesis or hereditary motor neuropathy are excluded.

**Conclusion:**

This rare case, together with three other literature reports by Sasaki (Intern Med 58(3):437–440, 2019), Sajra (Med Arh 61(1):52–53, 2007) and Matsubara et al., (J Neurol Neurosurg Psychiatry 57(5):640–642, 1994). suggests that Klinefelter syndrome can be associated with spastic paraparesis, besides the other various neuropsychiatric symptoms that are more commonly described.

## Introduction

Spastic paraparesis is characterized by progressive degeneration of the corticospinal tracts [1, 2]. There are several genetic and non-genetic and acquired causes. Klinefelter syndrome is the most common cause of primary hypogonadism with a broad clinical presentation [3, 4]. The association of Klinefelter syndrome and spastic paraparesis is rarely described, which makes this report valuable.

## Case report

A 61-year-old man sought advice in 2019 for a problem he had with gait for many years. At that time, he wore an neurostimulator for chronic lower back pain. Other relevant medical history include a car accident in 1997 and total prothesis of the left knee in 2016. He has high arched feet, for which he wears arch supports. His parents, who had died years before, and his two brothers were asymptomatic, although there is no certain information. The patient did describe high arched feet with his mother. The patient has no children because of infertility. He served in the army for four years after graduating and later worked as a trucker. Since 2003, he was on sick leave because of diffuse pain problems. The pain problems arose after his car accident.

Since 2019, he uses a scooter for long transports because of decreased strength in his legs. He also noticed weakness in his hands. He gets muscle cramps after 500 m of walking, but even in rest he has a deep muscle pain. At night he has clonus and tremors of the legs. Paresthesias became apparent in the thighs and also in the forearm and hands. There are intermittent complaints of urinary urge. The patient has no other neuropsychiatric problems.

Neurological examination shows a mild atrophy of the lower legs, high arched feet, hyperreflexia in the legs with unresponsive plantar reflexes and with presence of a clonus at the ankles. Furthermore there is an spastic gait.

Routine hematologic tests are normal. Vitamin, copper and ceruloplasmin, and long-chain fatty acid are normal. Antibodies to Treponema and Borrelia Burgdorferi are absent. Nerve conduction testing and myography are normal. The somatosensory evoked potentials in the legs is normal. MRI of the brain does not show any abnormalities. MRI of the spinal cord shows no extradural, intradural, and intramedullary abnormalities.

As the patient presented with a spastic paraparesis and, like his mother, with high arched feet, possibly hereditary, genetic testing was done. A panel for ataxia spasticity of 260 genes did not show pathogenic variants. The extensive molecular analysis for hereditary neuropathies of 211 genes was also negative. However, there were copy number variations indicating an possible extra sex chromosome. The presence of an additional X chromosome, confirmed with a microsatellite analysis, revealed the diagnosis of a Klinefelter syndrome in the patient.

A diagnosis of XXY Klinefelter associated spastic paraparesis was made.

## Discussion

Spastic paraparesis is characterized by progressive degeneration of the corticospinal tracts [1, 2], presenting with spasticity and weakness of the lower limbs. In most cases there is bladder involvement [1]. In other cases there are additional neurologic or systemic abnormalities such as peripheral neuropathy, MRI brain abnormalities, cognitive and hearing impairment, ataxia, distal muscle atrophy, visual loss, and epilepsy [5]. The progression is very variable. There are several genetic and non-genetic and acquired causes. Of the acquired causes there are the structural causes (extradural, intradural/extramedullary, and intramedullary), where high quality imaging of the spine is important [1], such as spinal cord compression, inflammatory causes such as multiple sclerosis, sarcoidosis, Sjögren syndrome, infections such as HIV and HTLV1 or metabolic diseases [6]. The genetic forms of spastic paraparesis have many different clinical presentations and also different genetic abnormalities. There are several genetic loci known and thus several forms are described with SPG3A and SPG4 the most common autosomal dominant forms, and SPG5 and SPG11 the most common autosomal recessive forms. X-linked heredity has also been described [2]. Further, many inborn errors of metabolism can present with late-onset spastic paraparesis [6].

The therapeutic approach of spastic paraparesis is symptomatic, because there is no disease-modifying treatment [7].

Klinefelter syndrome is the most common cause of primary hypogonadism, albeit with many undiagnosed cases because of its broad clinical presentation [3, 4]. The most typical karyotype is 47,XXY, but other karyotypes have been reported [3]. The typical presentation is the presence of hypospadias or micropenis, small testes, delayed puberty. The serum testosterone concentrations are low or low-normal and the serum gonadotropin concentration is high. Besides infertility, there is also an increased risk for learning and language disorders, metabolic syndrome and diabetes mellitus, cardiovascular events, thromboembolic disease, autoimmune disease, and certain cancers [4, 8]. The diagnosis is definite when there is at least one additional X chromosome with a Y chromosome.

The therapy is testosterone therapy when there are low serum testosterone concentrations or when there is normal serum testosterone but elevated serum luteinizing hormone (LH) [9].

After excluding genetic and acquired causes of spastic paraparesis as mentioned before, we confirmed a rare case of spastic paraparesis in our patient with Klinefelter syndrome, with an additional X chromosome. The patient had infertility, but no other typical physical or neuropsychiatric presentation of Klinefelter syndrome, such as an abnormal arm-span or mental retardation, which made this finding surprising.

There are three other reports described in the literature with Klinefelter associated spastic paraparesis. In the first report, the patient was affected with a mosaic form of Klinefelter syndrome (47, XXY/48, XXXY) and was suffering from spastic paraparesis as well as peripheral neuropathy [10]. In the second case the patient with Klinefelter syndrome had spastic paraparesis without peripheral neuropathy [11]. Finally, there is one report of two cases where patients had Klinefelter syndrome presenting with slowly progressive neurogenic muscle atrophy [12]. In our case, too, there was no clinical or electromyographic evidence for a peripheral neuropathy. The link between Klinefelter syndrome and spastic paraparesis remains rare, as for the link between Klinefelter syndrome and peripheral neuropathy [10, 11, 13, 14].

The mother of the patient had high arched feet as described by the patient. Unfortunately, his parents had already died, so no clinical examination or genetics of his parents could be obtained.

## Conclusion

We present a clinical case of chronic progressive spastic paraparesis and high arched feet in an infertile 61-year-old adult man with familial history of high arched feet. The symptomatology was attributed to the presence of a karyotype 47, XXY, in absence of other causes of spastic paraparesis after thorough investigations for other acquired or genetic etiologies.

## Data Availability

Not applicable.

## Declarations

### Author’s information

JDB is a member of the European Reference Network for Neuromuscular Diseases.
